# A medium-chain fatty acid analogue prevents hepatosteatosis and decreases inflammatory lipid metabolites in a murine model of parenteral nutrition-induced hepatosteatosis

**DOI:** 10.1371/journal.pone.0295244

**Published:** 2023-12-01

**Authors:** Bennet S. Cho, Scott C. Fligor, Gillian L. Fell, Jordan D. Secor, Savas T. Tsikis, Amy Pan, Lumeng J. Yu, Victoria H. Ko, Duy T. Dao, Lorenzo Anez-Bustillos, Thomas I. Hirsch, Jenny Lund, Arild C. Rustan, David A. Fraser, Kathleen M. Gura, Mark Puder

**Affiliations:** 1 Vascular Biology Program and Department of Surgery, Boston Children’s Hospital, Boston, Massachusetts, United States of America; 2 Harvard Medical School, Boston, Massachusetts, United States of America; 3 Department of Pharmacy, Section for Pharmacology and Pharmaceutical Biosciences, University of Oslo, Oslo, Norway; 4 NorthSea Therapeutics, Amsterdam, Netherlands; 5 Department of Pharmacy and the Division of Gastroenterology and Nutrition, Boston Children’s Hospital, Boston, Massachusetts, United States of America; University of Illinois, UNITED STATES

## Abstract

**Background:**

Parenteral (intravenous) nutrition is lifesaving for patients with intestinal failure, but long-term use of parenteral nutrition often leads to liver disease. SEFA-6179 is a synthetic medium-chain fatty acid analogue designed to target multiple fatty acid receptors regulating metabolic and inflammatory pathways. We hypothesized that SEFA-6179 would prevent hepatosteatosis and lipotoxicity in a murine model of parenteral nutrition-induced hepatosteatosis.

**Methods:**

Two *in vivo* experiments were conducted. In the first experiment, six-week-old male mice were provided an *ad lib* fat-free high carbohydrate diet (HCD) for 19 days with orogastric gavage of either fish oil, medium-chain triglycerides, or SEFA-6179 at a low (0.3mmol/kg) or high dose (0.6mmol/kg). In the second experiment, six-week-old mice were provided an *ad lib* fat-free high carbohydrate diet for 19 days with every other day tail vein injection of saline, soybean oil lipid emulsion, or fish oil lipid emulsion. Mice then received every other day orogastric gavage of medium-chain triglyceride vehicle or SEFA-6179 (0.6mmol/kg). Hepatosteatosis was assessed by a blinded pathologist using an established rodent steatosis score. Hepatic lipid metabolites were assessed using ultra-high-performance liquid chromatography-mass spectrometry. Effects of SEFA-6179 on fatty acid oxidation, lipogenesis, and fatty acid uptake in human liver cells were assessed *in vitro*.

**Results:**

In the first experiment, mice receiving the HCD with either saline or medium-chain triglyceride treatment developed macrovesicular steatosis, while mice receiving fish oil or SEFA-6179 retained normal liver histology. In the second experiment, mice receiving a high carbohydrate diet with intravenous saline or soybean oil lipid emulsion, along with medium chain triglyceride vehicle treatment, developed macrovescular steatosis. Treatment with SEFA-6179 prevented steatosis. In each experiment, SEFA-6179 treatment decreased arachidonic acid metabolites as well as key molecules (diacylglycerol, ceramides) involved in lipotoxicity. SEFA-6179 increased both β- and complete fatty oxidation in human liver cells, while having no impact on lipogenesis or fatty acid uptake.

**Conclusions:**

SEFA-6179 treatment prevented hepatosteatosis and decreased toxic lipid metabolites in a murine model of parenteral nutrition-induced hepatosteatosis. An increase in both β- and complete hepatic fatty acid oxidation may underlie the reduction in steatosis.

## Introduction

In patients with intestinal failure, or the inability to absorb sufficient nutrients or fluids from the gastrointestinal tract, intravenous administration of parenteral nutrition is a critical life-saving therapy. Unfortunately, long-term parenteral nutrition is associated with intestinal failure-associated liver disease (IFALD). IFALD is characterized initially by hepatic inflammation and cholestasis, particularly in infants, with histological features appearing as early as two weeks after parenteral nutrition initiation [1,2]. IFALD often progresses to a chronic steatosis and fibrosis-predominant phenotype, particularly in adults. In recent years, hepatoprotective lipid management and multidisciplinary care has reduced mortality and the need for transplantation, transforming IFALD into a chronic disease [3,4]. However, even with modern management, most children with intestinal failure still demonstrate liver enzyme elevation and abnormal liver histology on routine biopsy with uncertain long-term consequences, highlighting the need for new therapeutics to interrupt the development and progression of IFALD [5].

Fatty acid-based therapies are intriguing due to regulation of various metabolic and inflammatory pathways implicated in IFALD and other related liver diseases such as nonalcoholic steatohepatitis [6]. For patients with intestinal failure, who often have little remaining small bowel and decreased absorption capacity, long chain fatty acids may not be absorbed sufficiently to allow enteral administration. Long-chain fatty acid absorption requires emulsification, hydrolysis, active transport into enterocytes, and packaging into chylomicrons for lymphatic transport into the systemic circulation [7]. In contrast, medium-chain fatty acids (MCFAs) are easily absorbed in the intestine via passive diffusion and enter directly into the portal venous system, resulting in ‘liver-targeting’ [8]. Interestingly, it has been shown that decanoic acid, a MCFA that binds and activates peroxisome proliferator-activated receptor γ (PPARγ), reduces blood glucose without the typical weight gain seen with potent synthetic PPARγ agonists [9]. However, the efficacy of MCFAs as an oral drug in an unaltered form is likely limited by rapid hepatic metabolism in conjunction with a relatively low affinity for fatty acid sensitive receptors [10].

SEFA-6179 is a synthetic MCFA analogue with structural modifications designed to avoid rapid β-oxidation whilst maintaining the ability to bind and activate key fatty acid receptors. *In vitro*, SEFA-6179 is a peroxisome proliferator-activated receptor α (PPARα) partial agonist and peroxisome proliferator-activated receptor γ (PPARγ) full agonist [11]. We recently demonstrated that SEFA-6179 prevents cholestasis, steatosis, and fibrosis in a preterm piglet model of IFALD [11]. Here, we sought to specifically investigate the anti-steatotic effects of SEFA-6179 *in vitro* and *in vivo*. Our group has previously used a murine model of parenteral nutrition-induced hepatosteatosis that results in liver injury similar to the chronic IFALD phenotype seen in older children and adults [12]. We hypothesized that SEFA-6179 treatment would protect against steatosis and decrease inflammatory lipid metabolites in this model.

## Materials and methods

### Murine model of parenteral nutrition-induced hepatosteatosis

All animal experiments were approved by the Boston Children’s Hospital Institutional Animal Care and Use Committee in accordance with the NIH Animal Research Advisory Committee guidelines [13]. A murine model of parenteral nutrition-induced hepatosteatosis was utilized for these experiments [12]. Six week-old male C57BL/6J mice (Jackson Labs, Bar Harbor, ME) were provided either a standard chow diet or an enteral liquid fat-free high carbohydrate diet (HCD) equivalent to the parenteral nutrition administered to patients at Boston Children’s Hospital (20% dextrose, 2% amino acids, 30 mEq/L sodium, 20 mEq/L potassium, 15 mEq/L calcium, 10 mEq/L magnesium, 10 mmol/L phosphate, 36.67 mEq/L chloride, 19.4 mEq/L acetate, pediatric multivitamins and trace elements). Liver injury induced by an enteral HCD is similar to liver injury observed in rodent models using intravenous PN administration and avoids septic complications of catheter use. This allows for investigation of the metabolic liver consequences of continuous high carbohydrate provision (enterally or intravenously) without confounding from sepsis. Mice provided intravenous soybean oil lipid emulsion (SOLE) in addition to enteral HCD develop hepatosteatosis while intravenous fish oil lipid emulsion prevents steatosis [12]. This model has been used in multiple studies to recapitulate the effects of different intravenous lipid emulsions seen in patients [12,14,15].

Animals receiving HCD had no additional sources of nutrition or hydration. Animals were permitted *ad libitum* feeding of their respective diets for 19 days. Potential confounding was minimized by conducting the entirety of the experimental groups concurrently, identical housing, and use of vehicle controls within each experiment. The experiment was not blinded. After 19 days, animals were euthanized by carbon dioxide. Serum was collected by inferior vena cava puncture and sent to the laboratory at Boston Children’s Hospital for analysis of plasma biomarkers.

Two experiments were performed to evaluate SEFA-6179 in this model. The first experiment (experiment 1) consisted of enteral administration of SEFA-6179 via orogastric gavage in fat-free HCD-fed mice. The second experiment (experiment 2) tested enteral administration of SEFA-6179 in HCD-fed mice receiving either intravenous saline, SOLE, or fish oil lipid emulsion (FOLE).

#### Experiment 1

The purpose of the first experiment was to determine if SEFA-6179 prevented hepatosteatosis and biochemical liver injury in the absence of intravenous lipid emulsion treatment. Mice were randomized to one of six groups in Table 1 (8–12 mice/group). The sample size was determined based upon previous experiments using this model and the need for adequate tissue to conduct the analyses. One group received standard chow diet in pellet form (chow). The other five groups were fed a liquid HCD and received oral gavage treatment every other day of their respective treatments: saline, FOLE (Omegaven; Fresenius Kabi, Bad Homburg, Germany), medium-chain triglyceride oil vehicle (MCT) (Nestle HealthCare Nutrition, Florham Park, NJ), low dose SEFA-6179 (0.3 mmol/kg), or high dose SEFA-6179 (0.6 mmol/kg) (NorthSea Therapeutics, Amsterdam, Netherlands).

**Table 1:** Experiment 1 groups.

Group	Group Type	Diet	Oral Gavage
Chow	Normal Control	Chow	None
Saline	Negative Control	HCD	Saline
FOLE	Positive Control	HCD	Fish oil lipid emulsion
MCT	Vehicle Control	HCD	Medium-chain triglycerides
Low SEFA-6179	Treatment	HCD	SEFA-6179 (0.3mmol/kg)
High SEFA-6179	Treatment	HCD	SEFA-6179 (0.6mmol/kg)

Fat free liquid high carbohydrate diet (HCD).

The saline group served as a negative control and received 200 μL of saline, isovolumetric to MCT and SEFA-6179 groups. The MCT group served as a vehicle control group as MCT was used as a vehicle to dilute SEFA-6179. Mice in this group received 7.4 g of fat/kg body weight every other day of MCT. The FOLE group served as a positive control group. Mice in this group received 1.2 g fat/kg body weight every other day of FOLE as in our prior studies validating this model [12]. The remaining two experimental groups received oral gavage treatment every other day of SEFA-6179 at either a low dose (132 mg/kg or 0.3 mmol/kg) or high dose (264 mg/kg or 0.6 mmol/kg). Dosing was selected based on studies with a similar fatty acid analogue, icosabutate, and using interspecies allometric scaling as well as data from preliminary studies.

#### Experiment 2

The purpose of experiment 2 was to test the efficacy of SEFA-6179 in HCD-fed mice receiving intravenous lipids for 19 days, similar to patients receiving parenteral nutrition and a lipid emulsion. The high dose of 264 mg/kg body weight every other day, was selected based on the results of experiment 1. Mice were randomized to one of seven groups listed in Table 2 (n = 8-12/group). The HCD groups received every other day treatment with intravenous saline, SOLE, or FOLE via tail vein injection (2.4 g/kg/d). Additionally, mice received every other day oral gavage of either MCT vehicle or SEFA-6179 (264 mg/kg; 0.6 mmol/kg).

**Table 2:** Experiment 2 groups.

Group	Diet	Intravenous Injection	Oral Gavage
Chow	Chow	None	None
Saline / MCT	HCD	Saline	Medium-chain triglycerides
Saline / SEFA-6179	HCD	Saline	SEFA-6179 (0.6mmol/kg)
SOLE / MCT	HCD	Soybean oil lipid emulsion	Medium-chain triglycerides
SOLE / SEFA-6179	HCD	Soybean oil lipid emulsion	SEFA-6179 (0.6mmol/kg)
FOLE / MCT	HCD	Fish oil lipid emulsion	Medium-chain triglycerides
FOLE / SEFA-6179	HCD	Fish oil lipid emulsion	SEFA-6179 (0.6mmol/kg)

Fat free liquid high carbohydrate diet (HCD).

### Histology

Formalin-fixed paraffin-embedded liver samples were stained with hematoxylin and eosin (H&E) to assess hepatic architecture. Whole slide scanning (40x) was performed on an Aperio AT2 (Leica Biosystems) by HistoWiz, Inc. A blinded pathologist assessed each slide utilizing an established rodent nonalcoholic fatty liver disease scoring system [16]. Micro- and macrovesicular steatosis were scored independently from 0–3 based on the percentage of area affected. Hepatocellular hypertrophy was similarly scored. The three subscores were combined to create a single steatosis score (0–9).

### Liver lipidomic profile

Ultra-high-performance liquid chromatography–mass spectrometry (UHPLC-MS) was performed on frozen liver samples. Lipid metabolite analysis was performed in four groups for experiment 1 (chow control, MCT vehicle control, FOLE, and high dose SEFA-6179) but included all groups except FOLE/SEFA-6179 for experiment 2. Liver samples were sent to Owl Metabolomics (Bizkaia, Spain) for preparation and analysis. Proteins were precipitated from liver tissue (15 mg) with H_2_O (15:1, v/w), methanol containing the internal standards (50:1, v/w), chloroform:methanol (2:1) containing internal standards (4:1, v/w) and chloroform (40:1, v/w). Mixtures were homogenized and incubated at -20°C for 1 h. For fatty acyl, bile acid, steroid, and lysoglycerophospholipid profiling, samples were centrifuged at 18,000 x g for 15 min at 4°C, dried under vacuum, reconstituted in methanol, and resuspended with agitation for 20 min. Samples were centrifuged again for 5 min and transferred for UHPLC-MS analysis. For glycerolipid, cholesteryl ester, sphingolipid, and glycerophospholipid profiling, samples were mixed with ammonium hydroxide, vortexed, and incubated for 1 hour at -20°C. After centrifugation for 15 minutes, the organic phase was collected and dried under vacuum. Dried extracts were reconstituted in acetonitrile/isopropanol (1:1), resuspended with agitation for 10 min, centrifuged for 5 min, and transferred for UHPLC-MS analysis. All data from UHPLC-MS analysis was processed using the TargetLynx application manager for MassLynx 4.1 software (Waters Corp., Milford, USA). For identified metabolites, representative MS detection response curves were generated using an internal standard for each chemical class included in the analysis.

### In vitro experiments

Human hepatocytes (the hepatocyte-derived carcinoma cell line Huh7) were cultured in 96-well plates at 12000 cells/well. After 24 h the cells were either untreated, or pretreated with 0.1% DMSO (negative control), 5 μM SEFA-6179 (in DMSO), or 10 μM SEFA-6179 (in DMSO). After 48 h of pretreatment, cells received [1-^14^C]oleic acid (0.5 μCi/ml, 100 μmol/L) in DPBS supplemented with 10 mmol/L HEPES and CO_2_ was trapped over 4 h. Thereafter, the oleic acid media were saved to analyze acid-soluble metabolites (ASMs). ASMs mainly consist of tricarboxylic acid cycle metabolites and reflect incomplete fatty acid oxidation (β-oxidation) in the mitochondria. Then, cells were washed and lysed in 0.1 M NaOH. CO_2_ production (complete oxidation) and cell-associated (CA) radioactivity were assessed using a PerkinElmer 2450 MicroBeta2 scintillation counter. Protein levels in the lysate were measured by the Bio-Rad protein assay using a VICTOR X4 Multilabel Plate Reader. The sum of ^14^CO_2_, ASMs and remaining CA radioactivity was taken as a measurement of total cellular uptake of oleic acid: CO_2_ +ASM+CA.

For measurement of lipogenesis the cells were incubated in DPBS w/HEPES with [1-^14^C]acetate (2 μCi/mL, 100 μM) for 4 h and cell lysates were prepared by adding 0.1 M NaOH. Total lipids were isolated by filtration of the lysates through hydrophobic MultiScreen HTS plates (Millipore, Billerica, MA, US) and the radioactivity was determined by scintillation counting. Lipogenesis from acetate was normalized to lysate protein content measured by the Bio-Rad protein assay.

### Statistical methods

Serial body weights were compared after calculation of the area under the curve (AUC) for each mouse, adjusted for baseline weight. The area was calculated using the trapezoidal formula

$$\text{AUC}=\sum_{i}\sum_{j}{(T}_{ij+1}-T_{ij})\times (Y_{ij+1}+Y_{ij})$$

for mouse *i*, time *T*_*ij*_ (*j* = 0 to 19) and body weight *Y*_*ij*_. AUC was adjusted for baseline and interpreted as percent change from baseline by calculating *AUC*/(*Y*_0_/(*T*_19_ − *T*_0_)). Serum chemistries (experiment 1 only) and in vitro analyses were performed for n = 3 per group while lipid metabolite analysis was limited to 4 groups for experiment 1 (chow control, MCT vehicle control, FOLE positive control, and high SEFA-6179) but included all groups for experiment 2.

For analyses involving the full sample (body weight, organ weights and lipidomics), group comparisons were made by analysis of variance and described as mean ± SEM (standard error of mean). Adherence of each outcome to a normal distribution was assessed by Shapiro-Wilk test and visual inspection of a quantile-quantile plot. Given the limited sample sizes, we chose not to adjust for multiple comparisons. When the distribution of the outcome was skewed, the base 10 logarithm was used to transform the data. P values from the log-transformed analysis are shown with results on the original scale. For analysis involving serum chemistries and *in vitro* experiments where group sample sizes were n = 3 and n = 6, respectively, groups were compared by Kruskal-Wallis test. Pairwise comparisons were made by Dunn’s test unadjusted for multiple comparisons. Pathology was performed with n = 3 replicates per group for experiment 1 and n = 6 replicates per group for experiment 2. Due to the predominance of zeros and ties as well as the limited coverage over the score range of 0–9, pathology scores were dichotomized as 0–2 vs 3–9. Pairwise comparisons were made by Barnard’s test, which is more powerful than Fisher’s exact test for small sample sizes [17,18]. All data points were included in the analysis.

All tests were two-sided with P<0.05 deemed statistically significant. Analysis was performed with SAS version 9.4 (Cary, NC) and plots produced with GraphPad Prims 8.0.1 (GraphPad software, La Jolla, USA). Raw data are provided (S1 Dataset).

## Results

### Experiment 1—Evaluation of SEFA-6179 in the absence of intravenous lipid emulsion treatment

#### Body and organ weights

**Fig 1:**
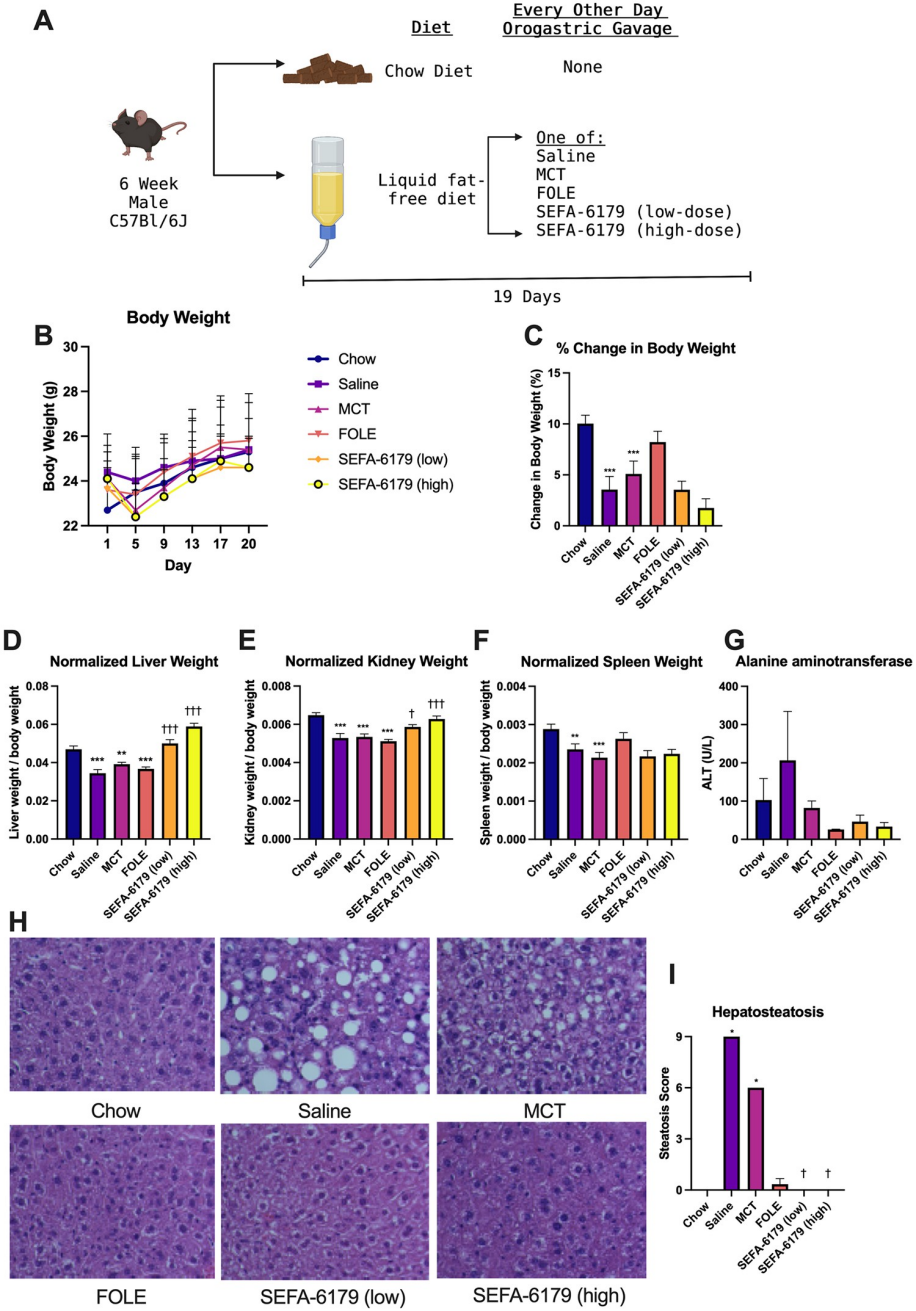
Provision of FOLE or SEFA-6179 to mice receiving a fat-free high carbohydrate diet prevents hepatosteatosis. 6-week-old male C57Bl6/J mice received an *ad lib* liquid fat-free high carbohydrate diet (HCD) with every other day orogastric gavage of saline, medium-chain triglyceride vehicle (MCT), fish oil lipid emulsion (FOLE), or SEFA-6179 (in MCT vehicle) at a low or high dose (A, created with biorender.com). Compared to chow, mice receiving saline or MCT lost weight (B, C); SEFA-6179 treatment did not change weight compared to MCT. SEFA-6179 treatment resulted in a dose-dependent increase in normalized liver weight and kidney weight, but not spleen weight, compared to MCT (D-F). Mice receiving the HCD developed increased serum alanine aminotransferase levels, with decreased levels in the FOLE and SEFA-6179 groups, although this did not reach statistical significance. Mice receiving saline or MCT developed macrovesicular steatosis; treatment with FOLE or SEFA-6179 (low or high dose) prevented steatosis (G, H). Mean ± SEM. Comparisons versus chow: * *P<*0.05 ** *P<*0.01 *** *P<*0.001. Comparisons versus MCT: † *P<*0.05 †† *P<*0.01 ††† *P<*0.001.

SEFA-6179 was well-tolerated with no adverse effects noted in animal behavior. The study design, change in body weight, and normalized organ weights are shown in Fig 1A–1F. All groups of mice gained weight over the 19-day course of the experiment (Fig 1B and 1C). Of note, all HCD groups experienced an early weight loss period prior to recovery and overall weight gain. This pattern of weight fluctuation is expected and characteristic of animals adapting to the HCD. Compared to the chow control group, mice in the saline and MCT groups gained less weight (6.1 ± 0.9 vs. 0.9 ± 0.8% *P* = 0.0007; 6.1 ± 0.9 vs. 0.4 ± 0.8%, *P* = 0.0002, respectively), although there was no difference between chow and FOLE (6.1 ± 0.9 vs. 4.0 ± 0.8%, *P =* 0.10). There was no difference in weight gain between the MCT and SEFA-6179 groups (low dose: 0.4 ± 0.8 vs. -0.3 ± 0.8, *P* = 0.53; high dose: 0.4 ± 0.8 vs. -1.5 ± 0.9, *P* = 0.11).

Compared to the chow control group, the HCD groups receiving saline, MCT, or FOLE all had decreased normalized liver weight (Fig 1D). Treatment with either low or high dose SEFA-6179 resulted in a dose-dependent increase in normalized liver weight from the MCT vehicle control (low dose: 0.039 ± 0.002 vs. 0.050 ± 0.002, *P*<0.0001; high dose: 0.039 ± 0.002 vs. 0.059 ± 0.002, *P*<0.0001). A similar relationship was observed in normalized kidney weight (Fig 1E). The HCD groups receiving saline, MCT, or FOLE demonstrated decreased normalized kidney weight compared to chow control. Treatment with low or high dose SEFA-6179 resulted in a dose-dependent increase in normalized kidney weight compared to MCT vehicle (low dose: 0.0053 ± 0.0002 vs. 0.0058 ± 0.0002, *P* = 0.0485; high dose: 0.0053 ± 0.0002 vs. 0.0063 ± 0.0002, *P*<0.0001). While treatment with HCD/saline or HCD/MCT resulted in decreased normalized spleen weight, compared to control, there was no difference between HCD/MCT and the two SEFA-6179 groups (Fig 1F; *P* = 0.60 for each).

#### Serum chemistries

HCD diets resulted in increases in serum alanine aminotransferase. However, these differences did not reach statistical significance (Fig 1G). There were no differences between the SEFA-6179 treatment groups and the MCT vehicle group.

#### Histology

On hematoxylin and eosin (H&E) staining, standard chow-fed controls demonstrated architecturally normal livers while HCD-fed mice receiving saline or MCT exhibited liver histology with diffuse macrovesicular steatosis (Fig 1H). Mice receiving FOLE maintained normal hepatic architecture. There was scarce microvesicular steatosis in the livers of mice receiving low dose SEFA-6179 while mice receiving high dose SEFA-6179 exhibited normal hepatic architecture. On pathologist scoring (Fig 1I), compared to chow, mice in the saline and MCT groups had an increased steatosis score (median 0 vs. 9, *P =* 0.03; median 0 vs. 6, *P =* 0.03 respectively). However, compared to the MCT vehicle, treatment with SEFA-6179 (low) and SEFA-6179 (high) decreased steatosis (median 6 vs. 0, *P* = 0.03 in each group).

#### Lipidomics

**Fig 2:**
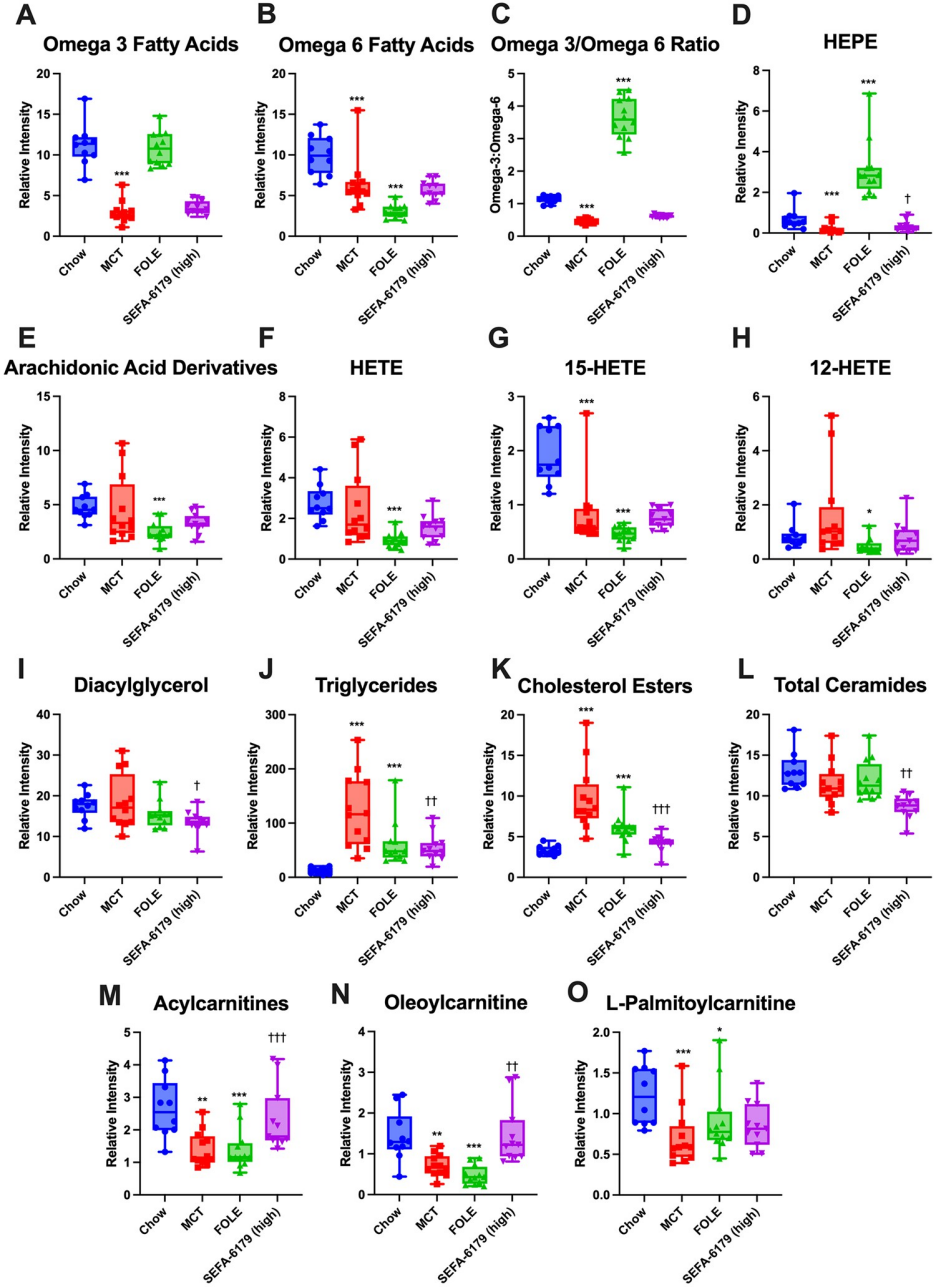
Treatment with SEFA-6179 in mice receiving a fat-free high carbohydrate diet decreases lipid metabolites involved in lipotoxicity including diacylglycerol, triglycerides, cholesterol, and ceramides. Ultra-high-performance liquid chromatography–mass spectrometry (UHPLC-MS) was performed on frozen liver samples. Lipid metabolite analysis was performed in four groups for experiment 1 (chow control, MCT vehicle control, FOLE, and high dose SEFA-6179). Mean ± SEM. Comparisons versus chow: * *P<*0.05 ** *P<*0.01 *** *P<*0.001. Comparison versus MCT: † *P<*0.05 †† *P<*0.01 ††† *P<*0.001.

Hepatic lipid metabolites involved in inflammatory signaling and fatty acid metabolism were analyzed (Fig 2). Pro-inflammatory lipid metabolites include omega-6 fatty acids and arachidonic acid derivatives including hydroxyeicosatetraenoic acids (HETEs), which are the precursors for leukotrienes and lipoxins. Ceramides are a type of sphingolipid that contribute to lipotoxicity in nonalcoholic fatty liver disease and nonalcoholic steatoheptatitis [19]. Anti-inflammatory metabolites include omega-3 fatty acids and hydroxyeicosapentanoic acids (HEPEs), which are eicosapentanoic acid-derived eicosanoids.

FOLE treatment, which consists of a high concentration of omega-3 fatty acids and minimal omega-6 fatty acids, resulted in predictable changes in lipid metabolites compared to the chow control. Compared to the chow control (presented as relative intensity ± SEM), FOLE treatment decreased omega-6 fatty acids (9.98 ± 0.66 vs. 3.06 ± 0.60, *P<*0.0001), arachidonic acid metabolites (4.8 ± 0.57 vs. 2.49 ± 0.52, *P =* 0.0004), total HETEs (2.76 ± 0.33 vs. 0.95 ± 0.30, *P<*0.0001), 15-HETE (1.91 ± 0.13 vs. 0.47 ± 0.12, *P<*0.0001), and 12-HETE (0.84 ± 0.29 vs. 0.48 ± 0.27, *P =* 0.04). FOLE treatment did not increase total omega-3 fatty acids (11.21 ± 0.57 vs. 10.84 ± 0.52, *P =* 0.83), but did increase HEPEs compared to chow (0.69 ± 0.25 vs. 3.11 ± 0.23, *P<*0.0001) and the omega-3:omega-6 ratio (1.13 ± 0.1 vs. 3.65 ± 0.09, *P<*0.0001). Finally, FOLE treatment did not alter the ceramide concentration compared to chow (13.07 ± 0.70 vs. 11.98 ± 0.64, *P =* 0.26).

Compared to chow-fed mice, MCT treatment, which does not contain omega-3 or omega-6 fatty acids, resulted in decreased omega-3 and omega-6 fatty acids (omega-3: 11.21 ± 0.57 vs. 2.94 ± 0.52, *P<*0.0001; omega-6: 9.98 ± 0.66 vs. 6.36 ± 0.60, *P =* 0.0002). The omega-3:omega-6 ratio was also decreased (1.13 ± 0.10 vs. 0.46 ± 0.09, *P<*0.0001). No differences were observed in arachidonic acid derivatives (4.80 ± 0.57 vs. 4.54 ± 0.52, *P =* 0.24) or ceramides (13.07 ± 0.70 vs. 11.49 ± 0.63, *P =* 0.10).

SEFA-6179 treatment was provided in a formulation combined with MCT. MCT was thus selected for the vehicle group. Compared to the MCT group, SEFA-6179 treatment did not affect omega-3 fatty acids, omega-6 fatty acids, omega-3:omega-6 ratio, arachidonic acid derivatives, HETE, 15-HETE, 12-HETE, or HEPE. Therefore, effects of SEFA-6179 on hepatic steatosis and inflammation are unrelated to altering the balance of omega-3 and omega-6 fatty acids. SEFA-6179 did, however, decrease ceramides compared to MCT (11.49 ± 0.64 vs. 8.67 ± 0.67, *P =* 0.004). SEFA-6179 treatment affected several metabolites involved in fatty acid oxidation and lipid trafficking. Compared to MCT vehicle, SEFA-6179 increased acylcarnitines (1.42 ± 0.22 vs. 2.32 ± 0.23, *P =* 0.003) including oleoylcarnitine (0.71 ± 0.14 vs. 1.47 ± 0.15, *P =* 0.0007), although not L-palmitoylcarnitine (0.70 ± 0.10 vs. 0.85 ± 0.11, *P =* 0.14), consistent with activation of fatty acid β-oxidation and the carnitine shuttle. SEFA-6179 treatment, compared to MCT vehicle, decreased cholesterol esters (9.69 ± 0.69 vs. 4.20 ± 0.72, *P<*0.0001), diacylglycerol (18.53 ± 1.26 vs. 13.46 ± 1.32, *P =* 0.01), and triglycerides (122.20 ± 12.43 vs. 55.23 ± 12.98, *P =* 0.002) consistent with improved lipid transport from the liver to peripheral tissues.

### Experiment 2—Evaluation of SEFA-6179 with parenteral lipid administration

#### Body and organ weights

**Fig 3:**
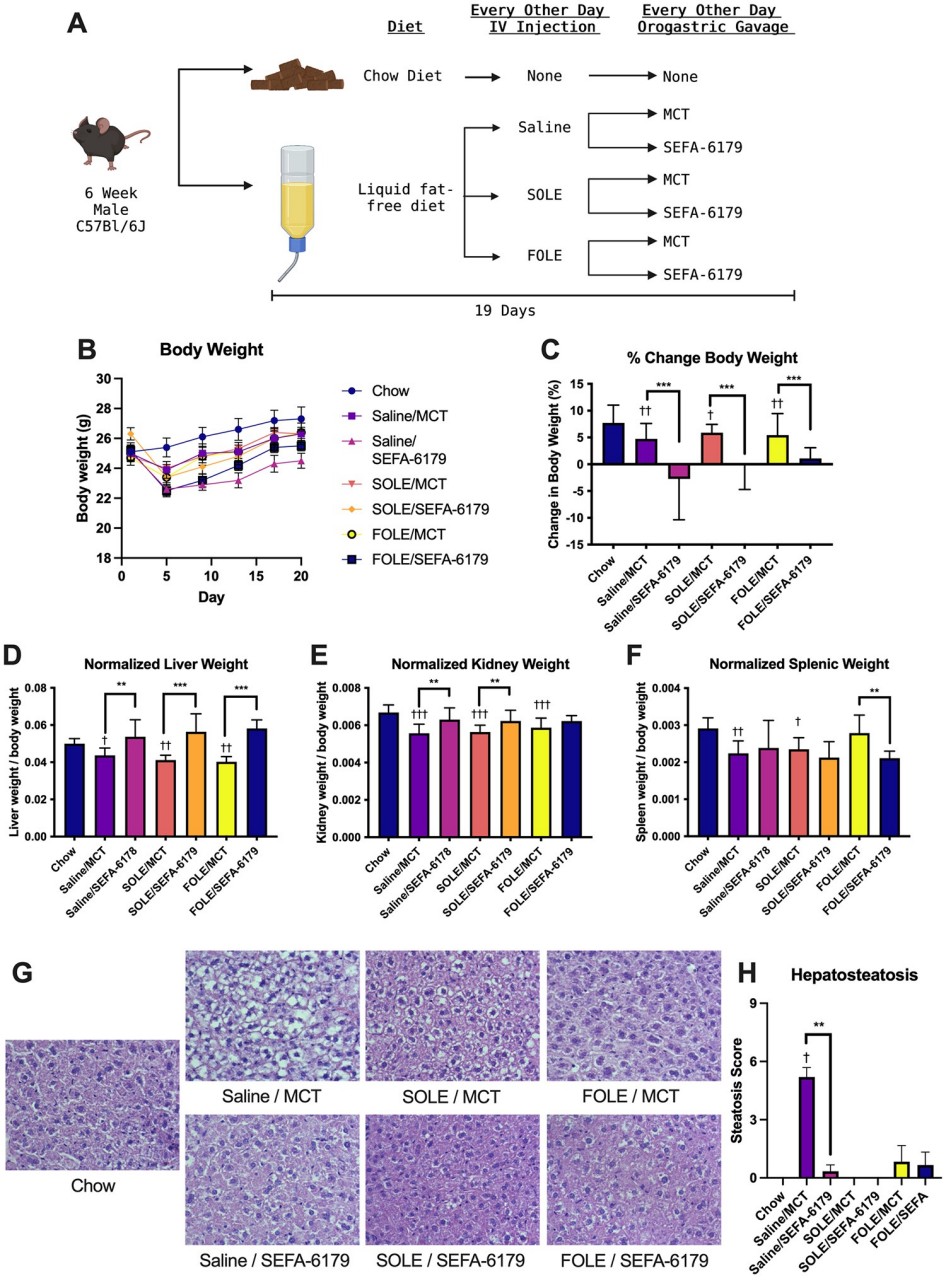
Treatment with SEFA-6179, compared to MCT vehicle, in mice receiving a high carbohydrate diet with intravenous saline or SOLE, results in prevention of steatosis. The study design is shown in A. Compared to chow, treatment with saline/MCT, SOLE/MCT, or FOLE/MCT resulted in decreased body weight (B, C). SEFA-6179 treatment, compared to MCT vehicle, decreased body weight further in each group. Compared to chow, treatment with saline/MCT, SOLE/MCT, or FOLE/MCT decreased normalized liver, kidney, and spleen weight (D-F). Treatment with SEFA-6179, compared to MCT, increased normalized liver and kidney weight. Treatment with saline/MCT resulted in steatosis, which was reversed with SEFA-6179 treatment (G, H). Mean ± SEM, Comparisons versus chow: † *P<*0.05 †† *P<*0.01 ††† *P<*0.001. SEFA-6179 compared to MCT within intravenous lipid emulsion groups marked by lines: * *P<*0.05 ** *P<*0.01 *** *P<*0.001.

The purpose of the second experiment was to investigate the impact of SEFA-6179 treatment in a murine model incorporating intravenous lipid emulsion therapy. The study design, change in body weight, and normalized organ weights are shown in Fig 3A–3F. Mice received an enteral HCD and intravenous lipid emulsion (SOLE, FOLE, or saline control) with oral gavage treatment of either MCT vehicle or SEFA-6179. Mice fed a standard enteral diet served as the control group (chow). Chow-fed mice gained more weight than the saline/MCT group (4.4 ± 0.9 vs. 0.2 ± 0.8%, P = 0.001), FOLE/MCT group (4.4 ± 0.9 vs. 0.7 ± 0.9%, *P =* 0.005), and SOLE/MCT group (4.4 ± 0.9 vs. 1.4 ± 0.8%, P = 0.01). Treatment with SEFA-6179 resulted in weight loss (saline/MCT vs. saline/SEFA-6179: 0.2 ± 0.8 vs. -6.2 ± 0.9%, P<0.0001; SOLE/MCT vs. SOLE/SEFA-6179: 1.4 ± 0.8 vs. -5.3 ± 0.8, P<0.0001; FOLE/MCT vs. FOLE/SEFA-6179: 0.7 ± 0.9 vs. -4.7 ± 0.8%, P = 0.0001). Compared to the chow-fed mice, mice in the saline/MCT, SOLE/MCT, and FOLE/MCT groups had decreased normalized liver weight. Treatment with SEFA-6179 resulted in increased normalized liver weight (saline/MCT vs. saline/SEFA-6179: 0.044 ± 0.002 vs. 0.054 ± 0.002, P = 0.004; SOLE/MCT vs. SOLE/SEFA-6179: 0.041 ± 0.002 vs. 0.056 ± 0.002, P<0.0001; FOLE/MCT vs. FOLE/SEFA-6179: 0.040 ± 0.002 vs. 0.058 ± 0.002, P<0.0001). A similar relationship was seen with kidney, but not spleen weight.

#### Histology

Representative histology is shown in Fig 3G. As above, on H&E staining, chow-fed mice exhibited normal hepatic architecture. HCD-fed mice receiving saline/MCT demonstrated diffuse macrovesicular steatosis while mice receiving saline/SEFA-6179 demonstrated normal hepatic architecture. Mice in the SOLE/MCT group demonstrated rare steatosis, while mice in the SOLE/SEFA-6179 group demonstrated normal hepatic architecture. Both FOLE groups (FOLE/MCT and FOLE/SEFA-6179) demonstrated preservation of normal hepatic architecture. On pathologist scoring (Fig 3H), compared to chow, mice in the saline/MCT group demonstrated increased steatosis (median 0 vs. 6, *P =* 0.002); compared to the saline/MCT group, treatment with saline/SEFA-6179 decreased steatosis (median 6 vs. 0, *P =* 0.001). There was no increased steatosis score in the SOLE/MCT or the FOLE/MCT group compared to chow.

#### Lipidomics

**Fig 4:**
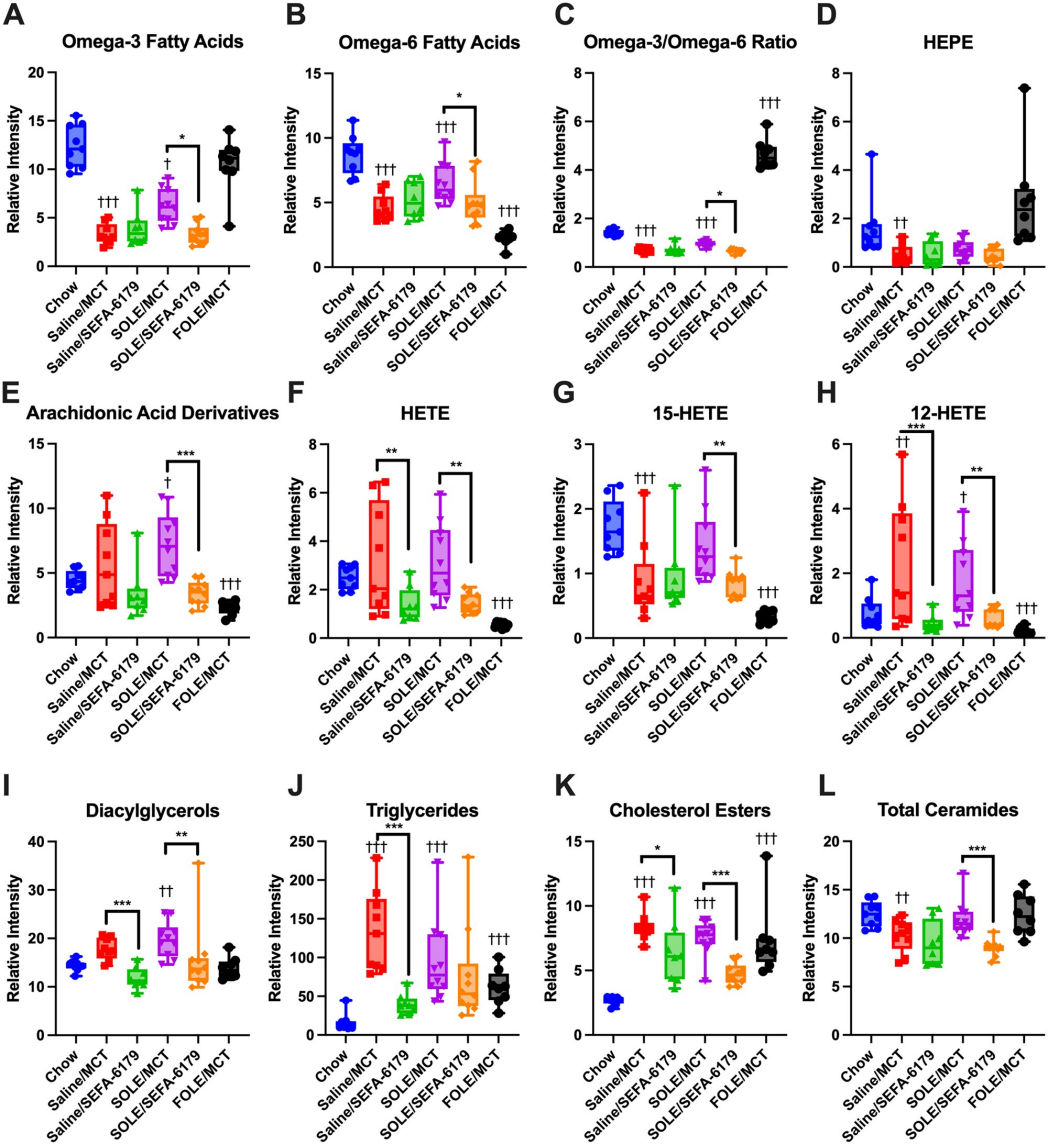
Treatment with SEFA-6179 in mice receiving a high carbohydrate diet (HCD) with intravenous saline or SOLE decreases lipid metabolites involved in lipotoxicity including diacylglycerol, triglycerides, cholesterol, and ceramides. Ultra-high-performance liquid chromatography–mass spectrometry (UHPLC-MS) was performed on frozen liver samples. Mean ± SEM. Comparisons versus chow: † *P<*0.05 †† *P<*0.01 ††† *P<*0.001. SEFA-6179 compared to MCT within intravenous treatment groups marked by lines: * *P<*0.05 ** *P<*0.01 *** *P<*0.001.

As in Experiment 1, lipid metabolites involved in inflammatory signaling and fatty acid metabolism were analyzed (Fig 4; data presented as relatively intensity ± SEM). The saline/MCT, SOLE/MCT, and FOLE/MCT groups were compared against the chow control. The SEFA-6179 treated groups were compared against their respective MCT vehicle controls groups (e.g., SOLE/MCT vs. SOLE/SEFA-6179).

Compared to the chow control group, treatment with SOLE/MCT decreased the content of omega-3 fatty acids (12.38 ± 0.62 vs. 6.17 ± 0.59, *P =* 0.02) and omega-6 fatty acids (8.74 ± 0.46 vs. 6.45 ± 0.43, *P =* 0.0006), but increased the content of arachidonic acid derivatives (4.49 ± 0.65 vs. 7.19 ± 0.62, *P =* 0.02) including 12-HETE (0.76 ± 0.32 vs. 1.73 ± 0.3, *P =* 0.02). Compared to chow, SOLE/MCT treatment also increased diacylglycerol (14.36 ± 1.29 vs. 19.73 ± 1.23, *P =* 0.002) and triglycerides (16.32 ± 14.41 vs. 94.81 ± 13.67, *P<*0.0001), but ceramides were similar (12.50 ± 0.58 vs. 11.95 ± 0.55, *P =* 0.49).

Treatment with SEFA-6179 (SOLE/MCT vs. SOLE/SEFA-6179) resulted in a mild decrease in omega-3 fatty acids (6.17 ± 0.59 vs. 3.28 ± 0.59, *P =* 0.02) and omega-6 fatty acids (6.45 ± 0.43 vs. 4.99 ± 0.43, *P =* 0.02). SEFA-6179 substantially decreased arachidonic acid derivatives in the SOLE groups (7.19 ± 0.62 vs. 3.43 ± 0.62, *P =* 0.0001) including total HETE (3.13 ± 0.38 vs. 1.44 ± 0.38, *P =* 0.001), 15-HETE (1.41 ± 0.14 vs. 0.84 ± 0.14, *P =* 0.008), and 12-HETE (1.73 ± 0.30 vs. 0.59 ± 0.30, *P =* 0.002). Finally, SOLE/SEFA-6179, compared to SOLE/MCT, decreased diacylglycerols (19.73 ± 1.23 vs. 15.60 ± 1.23, *P =* 0.003), triglycerides (94.81 ± 13.67 vs. 75.75 ± 13.67, *P =* 0.14), and ceramides (11.95 ± 0.55 vs. 8.99 ± 0.55, *P =* 0.0004).

### *In vitro* effects of SEFA-6179 on fatty acid oxidation and lipogenesis in human hepatocytes

**Fig 5:**
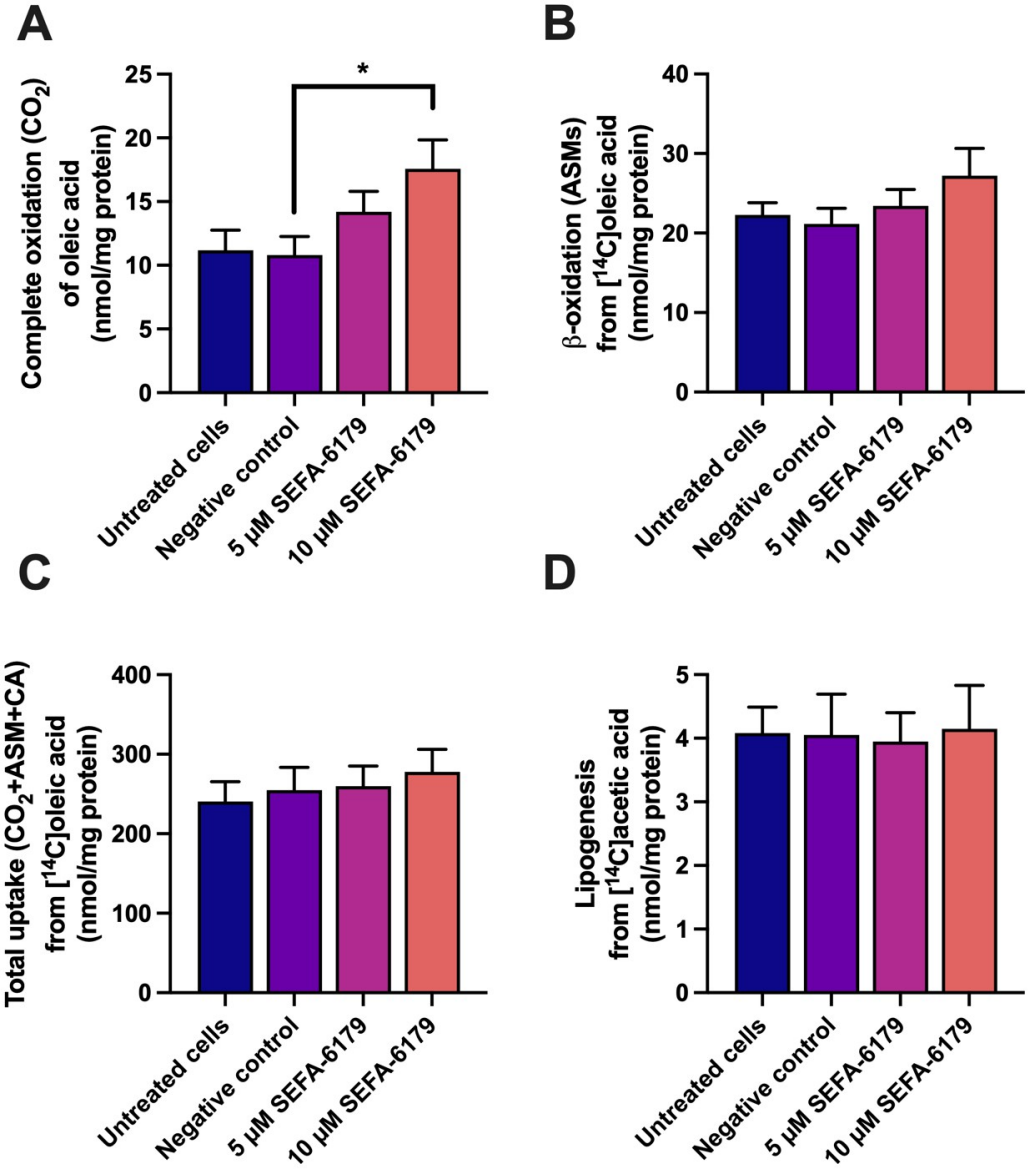
SEFA-6179 increases complete oxidation and β-oxidation of oleic acid in Huh7 cells, but does not affect oleic acid uptake or lipogenesis. Human hepatocytes (the hepatocyte-derived carcinoma cell line Huh7) were cultured in 96-well plates at 12000 cells/well (n = 6 wells/group in each experiment). After 24 h the cells were either untreated, or pretreated with 0.1% DMSO (negative control), 5 μM SEFA-6179 (in DMSO), or 10 μM SEFA-6179 (in DMSO). Treatment with SEFA-6179 increased complete oxidation of oleic acid and β-oxidation in a dose-dependent manner (A, B). Treatment with SEFA-6179 did not affect total oleic acid uptake or lipogenesis (C, D). Mean ± SEM. * *P<*0.05 vs. negative control.

We next sought to determine whether the anti-steatotic effects of SEFA-6179 observed in experiments 1 and 2 were the result of increased fatty acid oxidation or decreased lipogenesis utilizing human Huh7 cells. First oleic acid oxidation was assessed (Fig 5A–5C). Compared to DMSO vehicle-treated cells, treatment with SEFA-6179 increased both complete oleic acid oxidation and β-oxidation in a dose-dependent fashion (Complete oxidation: negative control vs. 10μM SEFA-6179 *P =* 0.02; β-oxidation: *P =* 0.13). Total cellular uptake of oleic acid was unaffected by pretreatment with SEFA-6179. Pretreatment with SEFA-6179 did not affect lipogenesis (Fig 5D).

## Discussion

Modern hepatoprotective management of intestinal failure has transitioned IFALD from a precipitous clinical course that often progressed to early liver transplant or death, to a chronic smoldering liver disease. Even with modern multidisciplinary management, most patients with intestinal failure have persistent abnormal liver histology on routine biopsy and up to 79% of pediatric intestinal failure patients on long-term parenteral nutrition have abnormal liver biomarkers [20, 21]. The long-term sequelae of chronic IFALD are currently unknown, as long-term survival for years or even decades was not possible until recent years with the use of fish-oil lipid emulsion and improvements in intestinal rehabilitation [4]. New therapies are necessary for prevention and treatment of IFALD as current therapeutic options are limited primarily to the avoidance of toxicity [22].

SEFA-6179 is a potential therapeutic under development for IFALD. In a preterm piglet model of IFALD, SEFA-6179 decreased hepatosteatosis; transcriptomic analysis showed activation of fatty acid oxidation pathways and inhibition of inflammatory pathways, consistent with *in vitro* PPARα/γ agonism [11]. In experiment 1, we utilized a model of steatotic liver injury driven by a liquid high carbohydrate diet and essential fatty acid deficiency. This model mirrors the metabolic component of IFALD driven by a continuous high glucose infusion rate. Despite the strong drivers of *de novo* lipogenesis inherent in the high carbohydrate diet, SEFA-6179 demonstrated robust anti-steatotic effects. *De novo* lipogenesis is a key component of dysregulated lipid handling in steatotic liver injury; in one study, hepatic *de novo* lipogenesis accounted for 38% of intrahepatic triglyceride content in patients with nonalcoholic fatty liver disease but only 11% of intrahepatic triglycerides in lean patients [23]. Mechanistically, SEFA-6179’s robust anti-steatotic effects are consistent with the metabolic effects of PPARα-mediated fatty acid oxidation and improved insulin sensitivity from PPARγ. This is supported by the *in vitro* increase in both β- and complete fatty acid oxidation in human liver cells, with no effect on *de novo* lipogenesis or oleic acid uptake. The increase in liver weight is also consistent with PPARα activation in rodents [24].

The effects of PPARγ activation on hepatosteatosis are complex. Hepatocyte-specific PPARγ activation is steatogenic: liver PPARγ overexpression increases steatosis, while liver PPARγ knockout in *ob/ob* mice decreases steatosis [25]. However, in multiple clinical trials, PPARγ agonists improve hepatic steatosis in patients with nonalcoholic steatohepatitis [26]. This apparent paradox occurs due to peripheral PPARγ activation that increases adipose tissue synthesis of adiponectin, which improves systemic and hepatic insulin sensitivity through AMPK activation, decreasing liver lipogenesis, as well as increasing fatty acid oxidation [27,28]. Plasma adiponectin increases levels of peripheral lipoprotein lipase and VLDL receptor, thus resulting in catabolism of plasma VLDL and triglycerols [29]. In experiment 2, we utilized a model of parenteral nutrition-induced hepatosteatosis, with a liquid high carbohydrate diet paired with intravenous lipid emulsion administration, providing a similar nutritional profile to humans receiving parenteral nutrition. The intravenous provision of a soybean oil lipid emulsion, comprised primarily of triacylglycerols, prevents essential fatty acid deficiency while also providing a plasma triacylglycerol bolus that must be cleared either through peripheral or hepatic uptake. Orogastric gavage treatment with SEFA-6179 maintained normal liver histology and shifted the lipid metabolome to an anti-inflammatory phenotype.

Omega-3 and omega-6 fatty acids include a diverse array of bioactive metabolites. Arachidonic acid and its derivatives, such as the HETE family of eicosanoids, are downstream to omega-6 fatty acids and are broadly considered pro-inflammatory, whereas HEPE family of eicosanoids are downstream to omega-3 fatty acids and considered anti-inflammatory [30,31]. FOLE is rich in omega-3 fatty acids with relatively lower concentrations of omega-6 fatty acids. SOLE predominantly contains omega-6 fatty acids. As expected, FOLE-treated mice exhibited high liver concentrations of omega-3 fatty acids and HEPE, and low concentrations of omega-6 fatty acids, arachidonic acid derivatives, and HETE. SOLE-treated mice exhibited the opposite with low levels of omega-3 fatty acids and HEPE and high concentrations of omega-6 fatty acids, arachidonic acid derivatives, and HETE. HETE was also elevated in saline-treated mice. Interestingly, SEFA-6179-treated mice had lower levels of arachidonic acid derivatives and HETE, suggesting that decreasing these inflammatory lipid mediators may play a role in preventing the development of hepatosteatosis.

SEFA-6179 treatment decreased ceramides and diacylglycerols, lipid metabolites that are implicated in lipotoxicity. Ceramides are sphingolipids that are cell membrane components, cell signaling mediators, and are important in inflammation, cell adhesion, migration, apoptosis, fibrosis, and response to stress [32]. Elevations in ceramides may result in oxidative stress, inflammation, and insulin resistance, leading to non-alcoholic fatty liver disease, cirrhosis, and hepatocellular carcinoma [19]. Reduction in ceramides may be another important anti-inflammatory and metabolic mechanism through which SEFA-6179 prevents hepatotoxicity. Diacylglycerol is an intermediate molecule that serves as a precursor to triacylglycerol used for storage or cellular membranes. However, in the setting of lipid overload, diacylglycerides are a key mediator of lipotoxicity [33]. Elevations in diacylglycerol are associated with the progression from nonalcoholic fatty liver disease to nonalcoholic hepatosteatosis and cirrhosis [34]. Furthermore, diacylglycerides, along with ceramides, are the putative drivers of hepatic insulin resistance in type 2 diabetes mellitus and hepatic lipid accumulation [35,36]. PPARγ agonists (such as SEFA-6179)–via adiponectin-mediated signaling–both mobilize fat from the liver to the peripheral tissues by increasing triacylglycerol uptake and inhibiting hepatic triacylglycerol synthesis, which thereby reduces liver diacylglycerols–the final precursor in triacylglycerol synthesis [35]. Mice in the SOLE/MCT group developed elevated diacylglycerol compared to chow control, consistent with the histologic steatosis and progression of IFALD. Treatment with SEFA-6179 reduced liver diacylglycerol in both the saline/MCT and SOLE/MCT groups, consistent with PPARγ agonism and is a potential key mechanism for preventing lipotoxicity.

This study has several limitations. IFALD is a heterogenous condition with an active inflammatory and cholestatic phenotype primarily in infants, and a chronic steato-fibrotic phenotype often seen in older children and adults. This model of murine parenteral nutrition-induced steatotic liver injury has been previously used to investigate the toxicity of intravenous lipid emulsions, with findings directly correlating between observed toxicity in this model with development of hepatosteatosis and liver enzyme elevation in humans. However, mice in this model retain the entirety of their bowel and do not develop cholestasis or histologic inflammation; thus, it may not reflect the inflammatory acute IFALD population. Notably, with the use of fish oil lipid emulsion and hepatoprotective management, the acute IFALD phenotype has become far less common than the chronic hepatosteatosis and liver enzyme elevation consistent with smoldering liver disease. In both experiments, the absence of long chain polyunsaturated fatty acids in the control diet may have caused essentially fatty acid deficiency, driving hepatosteatosis beyond that from the high carbohydrate diet. However, the profound anti-steatotic effects of SEFA-6179 even with the multiple strong drivers of *de novo* lipogenesis only further highlights the anti-steatotic effects of SEFA-6179. Mice also have different expression of SEFA-6179’s target receptors, with PPARα expressed at substantially higher levels in mice livers than in humans. Previous murine studies have demonstrated that liver hypertrophy occurs with PPARα agonism in mice, which does not correlate to observed toxicity in humans [24]. Mice receiving SEFA-6179 treatment in experiment 2 lost weight compared to MCT vehicle, which may be due to increased fat oxidation–both in visceral and peripheral tissue. Clinical trials in humans, especially in the neonate, should closely monitor for excessive weight loss.

## Conclusions

SEFA-6179 prevented hepatosteatosis and decreased inflammatory lipid metabolites in a murine model of parenteral nutrition-induced hepatosteatosis. *In vitro* findings suggest the improvement in steatosis is driven by up-regulation of both β- and complete fatty acid oxidation, without directly impacting lipogenesis. In addition, SEFA-6179 treatment decreased pro-inflammatory lipid metabolites and molecules implicated in lipotoxicity. SEFA-6179 may prove particularly valuable for preventing progression or even reversing the smoldering liver injury in the substantial chronic IFALD population with steatosis.

## Supporting information

S1 Dataset Raw data file. (XLSX)
